# Blocked by Gender: Disparities in COVID19 infection detection in Tamil Nadu, India

**DOI:** 10.3389/fpubh.2022.966490

**Published:** 2022-09-30

**Authors:** Tannistha Samanta, Kaushik Gopalan, Tanmay Devi

**Affiliations:** ^1^Department of Sociology, FLAME University, Pune, Maharashtra, India; ^2^Department of Computer Science, FLAME University, Pune, Maharashtra, India; ^3^Department of Social Sciences, Rice University, Houston, TX, United States

**Keywords:** social determinants of health, infection, gender, India, COVID-19

## Abstract

Globally, a gender gap in COVID-19 has been noted with men reporting higher share of both morbidity and deaths compared to women. While the gender gap in fatalities has been similar across the globe, there have been interesting disparities in the detection of COVID-19 cases in men and women. While wealthier, more developed nations have generally seen similar case detection in men and women, LMICs especially in Asia have seen far greater proportion of COVID-19 cases among men than women. We utilize age and sex-disaggregated data from the southern Indian state of Tamil Nadu across two waves of the pandemic (May 2020 – Nov 2020, and March 2021, to June 2021) and find that there were only ~70% as many detected COVID-19 cases among women as there were among men. Our initial reading suggested that this might be a protective effect of lower labor force participation rates among women across much of South Asia. However, subsequent sero-prevalence results from Tamil Nadu conducted on October-November 2020, and June-July, 2021 suggest that infection incidence has been similar among men and women; as is the case in countries with better health infrastructure. This empirical puzzle suggests that reduced case detection among women cannot be immediately associated with limited public exposure, but rather evidence of a chronic neglect of women in healthcare access. Overall, we contend that an attention to the gender context holds promise to effective interventions in detection and prevention that goes beyond the traditional epidemiological logic of diseases.

## Introduction

That gender is an important axis of inequality is well-documented in the public health scholarship globally (1). To be sure, health scholarship has consistently shown that women and girls make comparatively fewer gains in health care than men and boys across similar age and social registers in most societies. However, this empirical narrative was shifted in the early months of the COVID-19 pandemic when global data revealed that men were 2.5 times more likely to be infected and are also 2.4 times more at risk of dying from COVID-19 than women (2). For example, the Global Health 50/50 repository demonstrated significant gender gaps in infections and deaths, where men seemed to fare worse in both counts. Since then, a considerable body of scholarship has attempted to explain this variation by privileging lifestyle and socio-economic factors (e.g., labor) and critiquing the biologically deterministic way of explaining disease risk (3, 4). Put simply, in societies that are governed by pervasive gender norms, the social realities of men and women are vastly different affecting their social and health outcomes. Or as a noted medical anthropologist, Paul Farmer, explains with the notion “the social production of disease” (p. 261) emphasizing how social and economic positioning produce gendered risk in epidemics and infectious disease outbreaks (4). Of all factors, the gender inequality in the labor force, finds particular attention among experts attempting to explain the variation in infection and mortality due to COVID-19 exposure. For instance, Adams showed that the percentage of female deaths due to COVID-19 were higher in countries that also have a higher proportion of women in the full-time workforce (5). In another study, Lewandowski and colleagues argue that when women work, they are largely concentrated in sectors where workplace interactions are higher (e.g., care, hospitality and education) and so is the exposure to the contagion (6). This study is significant since it undergirds the importance of labor market segregation in explaining disease risk. Finally, based on the case of Belgium that reported one of the highest rates of COVID-19 infections among women in the early months of the pandemic, Giscard Assoumou Ella argues how women's greater mobility outside home served as a potent route for infections as women traveled for work and family reasons (7). Yet again, authors have also attributed the difference in mortality as an outcome of underreporting bias against women and overall female neglect in matters of health and well-being even in industrialized countries (3). While causality cannot be conclusively ascertained given the evolving nature of the pandemic and the data, it is clear from these studies that disease risk among women is often tied to labor, mobility and the overall gender context.

It is also clear that there is a strong empirical association between age and case fatality from COVID-19 globally (8). However, owing to data unavailability in low-to-middle income countries, authors have contended how meaningful analysis remains limited in terms of guiding interventions that are age and context sensitive (9). We address this empirical lament and utilize age and sex-disaggregated data from the southern Indian state of Tamil Nadu, across two waves of COVID-19 pandemic, to show how age and gender intersect to create paradoxes in infection incidence.

## Data and Methods

Data for this study comes from several sources. We rely on the daily Media bulletins put out by the Health and Family Welfare Department of the Government of Tamil Nadu (TN) (https://stopcorona.tn.gov.in/daily-bulletin/). The media bulletins are provided in PDF format, and the text content from these files using the *pdfminer* package in the Python programming language is extracted. Using an automated program appropriate keyword searches (https://github.com/kaubega/tn_scraping), we retrieve the cumulative caseload for Males and Females separately in the age groups 0–12, 13–60, and 60+ from the daily bulletins.

For the purpose of this study, we extracted the data from the TN for May 16, 2020 – June 30, 2021. We were able to process 407 daily bulletins out of the 410 days in this time period; the rest were either unavailable on the website or were formatted such that our software was unable to process them. Further, we analyze infection statistics separately for the 1st and 2nd COVID-19 waves that occurred in India. For our study, we define the 1st wave as having occurred in TN from May 16, 2020 – Nov 15, 2020, and the 2nd wave as having occurred between March 15, 2021, to June 30, 2021.

**Figure 1:**
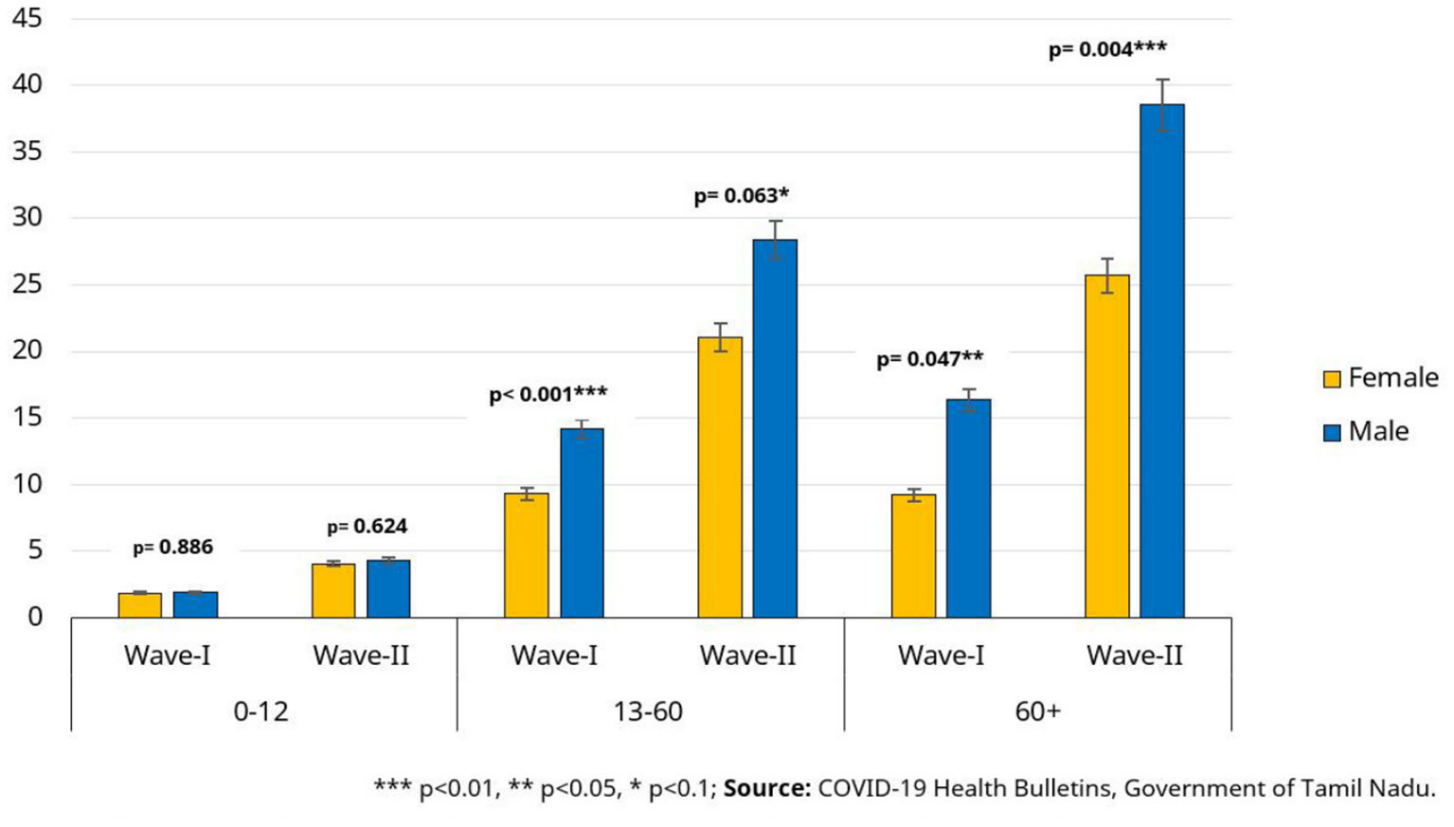
Confirmed cases per 1000 individuals (naive attack rate) for Tamil Nadu.

We examine the daily caseload data to analyze the spread of COVID-19 in different demographic groups. The TN media reports provide the number of detected infections in the 0–12, 13–60, and 60+ age groups, separately for males and females. In order to compare the extent of infection spread in the different demographic groups, detected infections are divided by the population of the respective demographic group as per Census 2011 to derive a “naive attack rate.” We observe substantial gender differences in the attack rates among adults, as shown in Figure 1. In Wave-1, there were 14.2 infections detected per 1,000 individuals aged 13 to 60 among men, compared to 9.3 per 1,000 individuals among women. This gender-gap favoring women is somewhat lower in Wave-2 in the 13–60 age group (and statistically significant only to 90% confidence); however, the naive attack rate is still ~35% higher among men. The gender difference is even greater in the older (60+ years) age group. The detected infections are ~75% higher among men in Wave-1 and 50% higher in Wave-2. This difference is however not observed among children.

While the confirmed COVID-19 case reports from Tamil Nadu suggest that men were infected at highly greater rates than women, multiple sero-prevalence surveys conducted in Tamil Nadu paint a different picture. Multiple rounds of the sero-prevalence surveys [e.g., see Table 2 of Selvavinayagam et al. (10)] suggest that infection incidence among men and women was similar after both Wave-I and Wave-II in Tamil Nadu. The sero-prevalence surveys were conducted on October-November, 2020 and June-July, 2021 respectively. We discuss this contradiction in detail in the discussion section.

## Discussion

As the COVID-19 pandemic continues to disrupt healthcare systems and lives, there is a growing recognition that availability of quality data on infections, fatalities and socio-demographic parameters of health remain a challenge in the middle-to-low income countries. To address this empirical dilemma, we used data from a southern Indian state that has meticulously published age and sex-disaggregated data on infections and fatalities since the onset of the pandemic in March 2020. We summarized key commonalities and differences in infections and fatality rates among men and women with a particular attention to older adults in the subsequent waves of the pandemic in India. Although, statistical estimates of infection or fatality antecedents were not possible to be modeled due to the limited nature of the data, this empirical summary allowed us to reflect on the intersection of age and the gender context, while reflecting in the paradoxical vulnerabilities of women and men.

One pathway that has been known to explain gender differentials in disease and mortality risks is labor force participation. Building on this line of inquiry, we contended that patriarchal ideologies that are known to restrict women's social and economic opportunities, unwittingly offer protection for older women from disease risk through restricted mobility. As such, the complex nexus between mobility and gender has been variously studied in the social sciences including those that focus on social norms as well as built environments (e.g., infrastructure and transportation). Feminist research on women's mobility patterns has shown that the claim “how people move (where, how fast, how often) is demonstrably gendered” (11) and perhaps age-coded, holds true for both the developing and the developed contexts (12–14). For example, in the Indian context, Lei et al. (15) show that in a context where labor mobility for women is deeply governed by gender attitudes and domestic obligations, transportation improves women's chances of non-farm job opportunities. In particular, they argue that road access and bus frequency can not only increase women's non-farm employment but can also enhance their bargaining power and autonomy to make decisions about their own health. In other words, one could argue that by staying at home women “bargained” with the patriarchal norms and social constraints over mobility, thereby optimizing their chances of reduced disease risk (16).

However, this line of reasoning is refuted by the sero-prevalence surveys conducted in Tamil Nadu that show similar levels of infections among men and women at all stages of the pandemic. This suggests that instead of a patriarchal “bargain” (noted earlier) that unwittingly protects women from infection, the gender gap in confirmed cases is caused by gender biases in testing for COVID-19 infection. We must, however, include the caveat here that sero-prevalence surveys routinely assign different weights to demographic groups in order to normalize the proportion of observations from each group. We do not have access to the specific weights used in the Tamil Nadu sero-survey and thus must allow for the possibility that statistical normalization might have some role in bridging the gender gap in the infection incidence. Despite this uncertainty, a gender bias in testing would not come as a surprise given a persistent female neglect in healthcare access and treatment is well-documented in middle to low-income countries globally and particularly in India. Demographers and public health experts have shown that despite overall advances in healthcare and rising levels of women's education and employment in India, female disadvantage in terms of (excess) mortality, neglect and discrimination continues throughout the life-course (17–19). Specifically, in terms of health access and outcomes, gender disparity has been remarkably stable in India with significant gradients by age and income (20–23). For example, in one estimate, out of 2,37,7028 outpatient visits, the authors calculated the overall sex ratio to be 1.69 male to one female visit (an equivalent of 4,02,722 missing female outpatient visits from four selected states in India) (21). As such, studies from other contexts have also emphasized how the pandemic has expanded the gender disparity in health. In their scoping review, Connor and colleagues report how the effects of heightened gendered disparity is felt more acutely among women vulnerable to poverty, IPV and racism in the United States (24). Specifically, the authors show that caregivers (who are typically women) have an increased exposure risk of contracting the infection while elevating the overall levels of multifactorial stress. Closer to home, feminist economist, Bina Agarwal's plea to understand the pandemic-led complex indirect gender effects on women is significant. She notes how preexisting gender inequalities and social norms can exacerbate unequal burdens of health and hunger, asset losses and abandonment of women and girls due to poverty (25). These household level disadvantages puts women at a higher risk since Indian women are known to have a higher incidence of comorbidities-malnourishment and anemia- and persistently lower levels of treatment seeking behaviors even when they carried higher burdens of multiple morbidities [see for example, Sandeep et al. (26)]. Notably, a persistent cultural regime of son-preference motived by social scripts that restrict women's economic and social freedoms with socio-legal implications (e.g., inheritance rules, remarriage laws), women continue to make losses in health and well-being throughout their life course.

Taken together, the COVID-19 data from Tamil Nadu thus tell us two contradictory stories: (a) the purported gender gap in the confirmed cases suggest a patriarchal “bargain” that protects women from infection as a consequence of their reduced mobility and (b) the sero-prevalence survey data that suggests that this gender gap may be an empirical illusion caused by systemic gender biases in COVID-19 infection testing. Given the history of persistent female neglect in healthcare in low-income, resource-constrained contexts, we believe that the second story is closer to the truth. In doing so, we address the plea of bridging the gap between feminist frameworks and empirical data. We hope this perspective piece offers an useful starting point to create synergies between evidence gathering, practice and research.

## Data availability statement

The original contributions presented in the study are included in the article/supplementary material, further inquiries can be directed to the corresponding author/s.

## Author contributions

TS contributed to the conceptualization and writing of the paper. KG contributed to data analysis and writing. TD contributed to data analysis. All authors contributed to the article and approved the submitted version.

## Conflict of interest

The authors declare that the research was conducted in the absence of any commercial or financial relationships that could be construed as a potential conflict of interest.

## Publisher's note

All claims expressed in this article are solely those of the authors and do not necessarily represent those of their affiliated organizations, or those of the publisher, the editors and the reviewers. Any product that may be evaluated in this article, or claim that may be made by its manufacturer, is not guaranteed or endorsed by the publisher.
